# Magnetoencephalography in Preoperative Epileptic Foci Localization: Enlightenment from Cognitive Studies

**DOI:** 10.3389/fncom.2017.00058

**Published:** 2017-06-28

**Authors:** Qian Wang, Pengfei Teng, Guoming Luan

**Affiliations:** ^1^Beijing Key Laboratory of Epilepsy, Sanbo Brain Hospital, Capital Medical UniversityBeijing, China; ^2^Department of Neurosurgery, Epilepsy Center, Sanbo Brain Hospital, Capital Medical UniversityBeijing, China; ^3^Beijing Institute for Brain Disorders, Capital Medical UniversityBeijing, China

**Keywords:** magnetoencephalographic (MEG), intracranial electroencephalogram (iEEG), distributed source modeling (DSM), resting-state, functional connectivity

## Abstract

Over 30% epileptic patients are refractory to medication, who are amenable to neurosurgical treatment. Non-invasive brain imaging technologies including video-electroencephalogram (EEG), magnetic resonance imaging (MRI), and magnetoencephalography (MEG) are widely used in presurgical assessment of epileptic patients. This review mainly discussed the current development of clinical MEG imaging as a diagnose approach, and its correlations with the golden standard intracranial electroencephalogram (iEEG). More importantly, this review discussed the possible applications of functional networks in preoperative epileptic foci localization in future studies.

## Introduction

Epilepsy is defined as a brain disorder characterized predominantly by recurrent and unpredictable interruptions of normal brain function resulting from abnormal and excessive neuronal discharges (Fisher et al., 2005). Approximately a third of epileptic patients are refractory to medication, who are amenable to neurosurgical treatment if evidences clarify focal network underlying the epilepsy (Jobst and Cascino, 2015). The main purpose of presurgery evaluation is to localize the epileptic foci in order to direct a precise surgery plan for an individual patient. Various of non-invasive brain imaging technologies including video-electroencephalogram (EEG), magnetic resonance imaging (MRI), positron emission tomography (PET), single photon emission computerized tomography (SPECT), and magnetoencephalography (MEG) play crucial roles in presurgical assessments of epileptic patients (Duncan et al., 2016). However, when the non-invasive outcomes are inconsistent, intracranial-electroencephalogram (iEEG), which is the current “golden standard” for localization of seizures, is needed to be conducted to confirm the involvement of suspected brain regions.

Among those non-invasive technologies, MEG has held the promise for localizing epileptic zones for its predictions of favorable post-surgical outcomes (Zhang et al., 2011; Englot et al., 2015b) and concordance with iEEG results (Almubarak et al., 2014; Grova et al., 2016; Murakami et al., 2016). One of the main advantages of MEG is its high spatial and temporal accuracy. By detecting the changes of magnetic fields produced by the electrical activities of neurons, epileptic spikes could be observed in MEG within only 4 cm^2^ cortical generators (Mikuni et al., 1997; Oishi et al., 2002; Shigeto et al., 2002). While a minimum area of 10 cm^2^ was required to detect spikes in scalp EEG (Tao et al., 2007).

Although MEG has numerous advantages in epileptic foci detection, it still can't replace the iEEG as an independent modality in presurgical assessment (Duncan et al., 2016). The most common MEG application now is to guide implantation sites for intracranial recordings (Knowlton et al., 2009; Agirre-Arrizubieta et al., 2014). One of the most important limitations exist in clinical MEG studies is the immature source reconstructing algorithm which is still developing. In the past decade, both the quantity and quality of MEG studies have remarkable increased, this state of the art technique seems promising to reveal brain mechanism of cognitive processing (Baillet, 2017). Going deep into understanding of human's brain, a large number of new concepts and perspectives are emerging. How could we take advantage of the exciting progresses made in cognitive MEG studies? This review attempted to discuss the recent development of clinical MEG studies and its application in preoperative epileptic foci localization.

## Source reconstruction of meg data

Interictal epileptic discharge (IED), which represents the field summation of potentials from a population of pathologically synchronized bursting neurons, is generally considered as a robust biomarker that identifies the epileptic foci (Oishi et al., 2002, 2006). However, the raw MEG signals are magnetic fields outside the human scalp (Cohen, 1968). A combination of MEG signals and structural MRI scans is needed to estimate the location and intensity of IEDs sources inside the brain which is called source localization. After artifact rejection and noise definition, source localization processing could be generally divided into two steps: (1) head modeling which establishes the electromagnetic properties of the sensor array and estimations of the brain sources; (2) source modeling imaging which solves an inverse problem (Tadel et al., 2011; Baillet, 2017).

Reconstructing of current dipoles, which are convenient model equivalents to the post-synaptic electrophysiological activity of local neuronal assemblies, is the basic concept in MEG source estimation process (Mosher et al., 1992). Among the current studies, two main approaches have been well adopted: single equivalent current dipoles (sECD), where the position and amplitude of one sECD is estimated over relatively short time windows, and distributed source modeling (DSM), which attempts to explain a given observed magnetic field by the distribution of source intensities in the brain instead of a single source (Tadel et al., 2011; Baillet, 2017).

The most common algorithm of the epileptic foci localization from clinical MEG recordings is sECD (Bagic et al., 2011). A well trained magnetoencephalographer is needed to extracts tightness and orientation of the dipole clusters visually (Salayev et al., 2006). Ideally, sECD can localize at best the center of the spatially extended generators as a point source (Ebersole, 1997). A retrospective study used sECD method revealed that patients with tight cluster and stable orientation dipole could significantly predict a better outcomes after surgery (Murakami et al., 2016).

However, the practicability of sECD method is debated not only for the subjective selection of IED period, but also for the possibility to mislead the presence of large spatially extended generators (Kobayashi et al., 2005). The latter is sophisticated because the IEDs were sometimes not confined to only one area (Agirre-Arrizubieta et al., 2009).

In the past decade, DSM has been largely promoted in cognitive MEG studies based on the fact that multiple brain areas would activate simulatonesly when a participant is performing a certain task (Tadel et al., 2011). DSM has also been applied in presurgical MEG evaluation using the nonlinear Maximum Entropy on the Mean (MEM) algorithm (Chowdhury et al., 2013; Grova et al., 2016; Heers et al., 2016; Zerouali et al., 2016), which could better predict the cortical regions with IEDs compared with other distributed models (Heers et al., 2016).

## Correlation with intracranial data

To evaluate the effectiveness of epileptic foci localization in MEG, the agreement between the MEG and “gold standard” invasive EEG approaches, which mainly include electrocorticography (ECoG) and stereo-electroencephalogram (sEEG) should be considered. Although most studies focus on the evaluation consistency between MEG and ECoG (Oishi et al., 2002, 2006; Knowlton et al., 2009; Almubarak et al., 2014), more and more studies has paid attention to the correlations between MEG and sEEG (Bouet et al., 2012; Grova et al., 2016; Murakami et al., 2016), which is considered to be minimal invasive and more suitable for preoperative evaluation (Gonzalez-Martinez et al., 2013; Zhou et al., 2017).

The effectiveness of MEG is depends on the depths of IED sources. Previous studies showed that MEG could detect 50–90% of spikes detected on iEEG superficial cortex including the lateral temporal cortex (Shigeto et al., 2002; Oishi et al., 2006; Agirre-Arrizubieta et al., 2009), while it could detect about 50–63% iEEG spikes in mesial temporal structures (Santiuste et al., 2008). These results are in general acceptable for presurgical assessments. Recent studies have revealed that epileptic activity from the insula, which is far away from scalp, can also be detected by MEG (Heers et al., 2012; Mohamed et al., 2013).

The limitation of iEEG approaches is it can only monitor a portion of brain areas, while MEG can provide a broad view of whole head activities and therefore may aid the interpretation of iEEG data. Murakami et al. (2016) systematically examined the consistency between the interictal discharges in MEG with interictal and ictal discharges in sEEG in 50 retrospective cases. The results showed that the proportion of seizure-free after epileptic foci resection was significantly higher when sEEG completely sampled the area identified by MEG. This finding suggests that although the MEG is still unable to take the place of iEEG as the standard, more weight should be assigned to MEG outcomes in combination of multiple modalities in preoperative evaluation.

## Epilepsy as a network diseases

One of the core questions in cognitive neuroscience is how different brain areas cooperate during perceptual/cognitive processing (Bressler and Menon, 2010). This hypothesis could also applied when epilepsy is considered as a network diseases (Laufs, 2012). Recent studies suggested that the brain connectivity differences among individuals could effectively interpret the patients with differing surgical outcomes (Englot et al., 2015a; Munsell et al., 2015).

Traditional epileptic network is defined as discharge propagation network (Malinowska et al., 2014), which is correlated with patient surgical outcome (Tanaka et al., 2014). However, a portion of patients could not be captured epileptic discharges during MEG recording. Functional connectivity studies have been recently applied in epileptic patients based on the assumption that the normal functions of local networks are impaired in the foci areas. Evidence for altered focal functional connectivity in patients with temporal lobe epilepsy has been reported using resting-state functional MRI method (Haneef et al., 2012; Cataldi et al., 2013; Maccotta et al., 2013).

Unlike the extensive studies using function MRI modality, the resting-state MEG studies are rare. Krishnan et al. (2015) conducted a preliminary MEG study and suggested that accurate localization of the epileptogenic foci may be accomplished using non-invasive spontaneous resting-state signals without the need to capture IEDs. Zerouali et al. (2016) used resting-state MEG analysis to recognize insular epileptic foci and revealed that anterior and posterior sub-regions of the insula have distinct patterns of functional connectivity, which could further guide different surgery plans.

A combination of resting-state fMRI and MEG studies of functional connectivity could be adopted to investigate the pathophysiology of epileptic networks. However, whether there is potential benefit in prediction of epilepsy surgery outcome in individual level is not established yet. To achieve this goal, a large database of normal resting-state mapping should be established. With a temporally rich source of information on brain network dynamics, MEG was called upon to join in the Human Connectome Project (Larson-Prior et al., 2013). Three years later, Niso et al. (2015) published an open archive resting-state MEG data of 97 health participants, which mainly focus on the distributions of typical oscillation frequency bands (delta, theta, alpha, beta, and gamma). Interestingly, a previous study shown that coherent neural activity change in the beta band might be causally involved in epilepsy (Heers et al., 2014), suggesting an exciting practicability of this MEG normative repository in preoperative evaluation for individual epilepsy patient.

To sum up, with the development of cognitive neuroscience, our understanding of normal and abnormal functional mechanism of the brain will get better and better. Using advanced MEG analysis technology, objective biomarks to locate epileptic foci is likely to replace the traditional subjective judgments in the upcoming future.

## Author contributions

QW, PT, and GL all contributed to the writing and revision of this manuscript.

### Conflict of interest statement

The authors declare that the research was conducted in the absence of any commercial or financial relationships that could be construed as a potential conflict of interest.
